# The Development of a Novel qPCR Assay-Set for Identifying Fecal Contamination Originating from Domestic Fowls and Waterfowl in Israel

**DOI:** 10.3389/fmicb.2016.00145

**Published:** 2016-02-17

**Authors:** Shoshanit Ohad, Shifra Ben-Dor, Jaime Prilusky, Valeria Kravitz, Bareket Dassa, Vered Chalifa-Caspi, Yechezkel Kashi, Efrat Rorman

**Affiliations:** ^1^National Public Health Laboratory Tel Aviv, Ministry of HealthTel Aviv, Israel; ^2^Bioinformatics Unit, Department of Biological Services, Weizmann Institute of ScienceRehovot, Israel; ^3^Bioinformatics Core Facility, National Institute of Biotechnology in the Negev, Ben-Gurion University of the NegevBeer-Sheva, Israel; ^4^Faculty of Biotechnology and Food Engineering, Technion – Israel Institute of TechnologyHaifa, Israel

**Keywords:** birds, fecal contamination, HTS, MST, qPCR

## Abstract

The emerging microbial source tracking (MST) methodologies aim to identify fecal contamination originating from domestic and wild animals, and from humans. Avian MST is especially challenging, primarily because the Aves class includes both domesticated and wild species with highly diverse habitats and dietary characteristics. The quest for specific fecal bacterial MST markers can be difficult with respect to attaining sufficient assay sensitivity and specificity. The present study utilizes high throughput sequencing (HTS) to screen bacterial 16S rRNA genes from fecal samples collected from both domestic and wild avian species. Operational taxonomic unit (OTU) analysis was then performed, from which sequences were retained for downstream quantitative polymerase chain reaction (qPCR) marker development. Identification of unique avian host DNA sequences, absent in non-avian hosts, was then carried out using a dedicated database of bacterial 16S rRNA gene taken from the Ribosomal Database Project. Six qPCR assays were developed targeting the 16S rRNA gene of *Lactobacillus, Gallibacterium, Firmicutes, Fusobacteriaceae*, and other bacteria. Two assays (Av4143 and Av163) identified most of the avian fecal samples and demonstrated sensitivity values of 91 and 70%, respectively. The Av43 assay only identified droppings from battery hens and poultry, whereas each of the other three assays (Av24, Av13, and Av216) identified waterfowl species with lower sensitivities values. The development of an MST assay-panel, which includes both domestic and wild avian species, expands the currently known MST analysis capabilities for decoding fecal contamination.

## Introduction

Microbial source tracking (MST) methodologies (Stoeckel and Harwood, 2007; Gourmelon et al., 2010; Marti et al., 2013) improve microbial monitoring resolution of surface and underground water influenced by human and animal fecal contamination. MST facilitates analysis of fecal contamination sources and their impact in a given geographical area. Data evaluation can support environmental management decisions. For example, the efficacy of corrective actions (Converse et al., 2012) can be monitored with MST. Avian species are a known reservoir of diverse pathogens (VerCauteren et al., 2012; Sandor et al., 2014), and may contribute to local and global epidemiology.

The fecal microbiome is dynamic, influenced by age, nutrition, and health. The Aves class (Zhu et al., 2002; Scupham et al., 2008; Lu et al., 2009; Xenoulis et al., 2010), comprising both domestic and wild birds, demonstrates highly diverse habitats and dietary characteristics which challenge MST marker design and evaluation. These factors have a direct influence on assay sensitivity and specificity values, which are crucial for assay assessment, because they reflect true positive and true negative detection rates. Assay sensitivity indicates the incidence of the marker within the target host population, and assay specificity points to the exclusivity of the marker to the target host. MST markers (Shanks et al., 2008; Boehm et al., 2013; Reischer et al., 2013) can be highly sensitive and specific; serial MST markers for gulls (Sinigalliano et al., 2013), targeting the *Catellicoccus marimammalium* bacteria, demonstrated a high percent of sensitivity values ranging from 81 to 100%. In this publication, the original specificity of assays varied from 37 to 85%, and was increased to 86 to 96% based on the following calculations: standardization of data, interpretation detected not quantified (DNQ) results as true negative and pigeon results as true positive. Notably, testing pigeon droppings using the Gull quantitative polymerase chain reaction (qPCR) assays yielded amplification levels comparable to those found in gull droppings, and in selected cases, even higher. This finding emphasizes other aspects of the complexity of avian MST design as different birds share the same habitats/environmental niches and possess similar bacterial communities. Moreover, they can easily fly and change locations carrying bacteria to different geographic locations.

Molecular human MST markers target unique sequences in bacterial, viral, coliphage, and mitochondrial DNA. Human MST, based on qPCR or PCR, identifies bacterial DNA sequences affiliated with (Ahmed and Katouli, 2008) *Enterococcus faecium*, (Harwood et al., 2009) *Methanobrevibacter smithii*, (Balleste and Blanch, 2011) *Bifidobacterium dentium*. The 16S rRNA gene (Bernhard and Field, 2000; Layton et al., 2006; Haugland et al., 2010) is currently the most prominent amplification target among *Bacteroidales* assays. The 16S rRNA gene (Clarridge, 2004) is an important housekeeping gene composed of highly conserved sequences as well as variable regions. Targeting PCR to its conserved regions allow DNA amplification originating from a large range of prokaryotes, while the hyper-variable sequences can be used for discriminatory sequence analyses. The number of base substitutions in the 16S rRNA gene is relatively low in close related bacteria species presenting low discriminatory resolution at sub genus and species level. These features turned 16S rRNA gene into a prominent gene used in bacterial taxonomy and phylogeny. However, other genes such as (Yampara-Iquise et al., 2008) the *thetaiotaomicron*-specific α-1-6, mannanase, and (Shanks et al., 2009) *Bacteroidales*-like cell surface-associated genes have also been reported. Avian MST markers developed thus far, identify bacteria of various taxonomic groups, including (Shen et al., 2013) *Faecalibacterium*, (Green et al., 2012; Ryu et al., 2012) *C. marimammalium*, and (Weidhaas and Lipscomb, 2013; Ryu et al., 2014) *Brevibacterium* sp. 16S rRNA gene clone libraries (Lu et al., 2009) from geese droppings were used to explore microbial communities from these sources to design host-specific assays. Recently (Shen et al., 2013), *in silico* data mining focusing on *Faecalibacterium* 16S rRNA gene from various animal species recognized an intervening sequence unique to poultry. The assay also identified feces of poultry and turkey, but not goose or seagull.

Our goal was to screen fecal samples of different avian species, both domestic and wild (waterfowl), and to identify specific DNA sequences within each group. Avian species may have overlapping bacterial profiles. We attempted to reveal subtle variations using the same experimental setting simultaneously. This approach required massive data collection which was accomplished using high throughput sequencing (HTS).

## Materials and Methods

### Fecal Sample Collection and Sample Processing

A total of 213 fecal samples from humans (25 wastewater), domestic and wildlife animals (52 battery hen, 22 poultry, 12 turkey, 23 waterfowl, 40 bovine, 20 swine, 9 equine, 10 gazelle) were collected at diverse farms and at various geographic locations in Israel from April 2010 to August 2014. Samples of gazelle and waterfowl droppings were taken at a single location, and samples from other hosts were collected at several separate locations (the amount of which appears in brackets) as follows: urban wastewater treatment plants (5); battery hens (4 enclosures), poultry (2 enclosures), turkey (2 enclosures), bovine (5 cow sheds), swine: (2 pig sties), and equine (2 stables). Feces and dropping samples collected were as fresh as possible, and grab samplings of wastewater primary eﬄuents were carried out into sterile polypropylene tubes and containers, respectively. Samples were shipped to the laboratory on ice within up to 3 h and kept at -80°C until the DNA extraction step.

DNA extraction from all environmental samples was conducted using the PowerSoil DNA isolation kit (MoBio, Carlsbad, CA, USA), according to the protocol of the manufacturer. The quality and quantity of avian source DNA preparations were analyzed by NanoDrop ND 1000 UV spectrophotometer (Thermo Fisher Scientific, Inc., Vienna, Austria) and samples were kept at -20°C until use.

### High Throughput Sequencing

The HTS initial sample preparation step comprised two sequential PCR amplifications. First, a portion of 16S rRNA gene spanning V1–V3 regions was amplified by the TaKaRa Ex Taq polymerase (TAKARA Bio, Inc., Otsu, Japan), using the primers 27F, 5′-AGAGTTTGATCCTGGCTCAG-3′, and 518R, 5′-GTATTACCGCGGCTGCTGG-3′. PCR was conducted in a Biometra thermocycler (Biometra, Goettingen, Germany), under the following cycling conditions: an initial PCR step for 7 min at 95°C, followed by 35 cycles: 30 s at 95°C, 30 s at 55°C and 60 s at 72°C, and a final extension step at 72°C for 10 min. PCR products were column-purified by Wizard SV Gel and PCR Clean-up System (Promega, Mannheim, Germany). A second PCR amplification was carried out using primers of 27F and 518R fused to Titanium Primers: A, 5′-CGTATCGCCTCCCT CGCGCCATCAG-MID-27F -3′, and B, 5′-CTATGCGCCTTGC CAGCCCGCTCAG-MID-518R-3′, under the same cycling condition. The sequences of the six Multiplex Identifiers (MID) were as follows: turkey (MID1, ACGAGTGCGT); waterfowl 1 (MID2, ACGCTCGACA); waterfowl 2 (MID3, AGACGCACTC); stork (MID4, AGCACTGTAG): battery hen (MID5, ATCAGACACG); poultry (MID6: ATATCGCGAG). Amplification products of 650 bp length were extracted from 0.8% agarose gel using Wizard SV Gel and PCR Clean-up System (Promega, Mannheim, Germany). Quality assessment of PCR amplification products, 454 pyrosequencing library construction and sequencing were performed at DYN Labs Ltd. (Dyn labs, Caesarea, Israel). The samples were tested using HTS and named poultry, turkey, battery hen, waterfowl 1, waterfowl 2, and stork. The reads have been deposited in the SRA database (NCBI) accession number SRP065761 under the BioProject accession number PRJNA300726.

### High Throughput Sequencing Data Analysis

High-quality multiplex reads were processed using QIIME Version 1.5.0 (Caporaso et al., 2010), selecting minimum quality score >25, minimal length >200 bp, containing no ambiguous bases or mismatches in the primers. The workflow for *de novo* operational taxonomic units (OTUs) picking included clustering of all the sequences into OTUs, based on their sequence similarity using the uclust clustering algorithm (Edgar, 2010). A representative set of sequences for each OTU was selected for subsequent analyses, including a taxonomy assignment by the RDP-classifier Version 2.2, using Naive Bayes classification (Wang et al., 2007). Training reference sequence set used for the classifier was the Greengenes sequence database release 12_10 (McDonald et al., 2012). Sequence identities of 97, 95, and 80% were used to approximate the species, genus and family taxonomical levels respectively, as previously described (Kuczynski et al., 2011).

In order to identify OTUs exclusive to each sample, we selected OTUs with a minimum of eight reads which were assigned only to a single group out of the six samples. A representative sequence of each 97% sequence distance defined OTU was compared for similarities with sequence reads derived from the various samples, using BLAST algorithm (Altschul et al., 1990) version 2.2.28+, in order to verify that it uniquely matches only one sample (BLASTn, with a minimum e-value of 0.001). An additional BLAST search was performed with each selected read against the non- redundant database at NCBI to ensure species specificity.

### Database Construction

The database of bacterial 16S rRNA gene was taken from the RDP database (Cole et al., 2014) unaligned sequences in GenBank format, Release 11.1. The sequences were filtered for those whose source indicated feces (feature table tag isolation_source, search terms: feces, fecal, faeces, faecal, stool), were not from a viral or vertebrate source organism, and had a clearly indicated host species). On several entries the host species were automatically corrected to translate from common names to those listed in NCBI’s Taxonomy, e.g., the host swine replaced by *Sus scrofa* and the host dog replaced by *Canis lupus*. The final database is composed of 127,218 sequences, and is available upon request.

### Search for Unique Sequences

The sequences were input into the open access MST search server: http://mst.weizmann.ac.il. The input sequence was compared to the database fecal 16S rRNA gene (described above) with FASTA Version 36.3.6 (Pearson and Lipman, 1988), with the E-value set to 1e-10. Clustering on the hits of the similarity search was performed to reduce redundancy using CD-HIT Version 4.6 (Fu et al., 2012), with the following parameters changed from the defaults: -M 0 –T 2. The longest representative sequence was taken for each cluster. Sequences left after removing redundancy were aligned with Muscle Version 3.8.425 (Edgar, 2004), with the following parameters: -maxhours 1, -maxiters 1, -diags. The alignment was trimmed to the region that overlapped with the input sequence. In order to find unique sequences that could be taken as probes, a sliding window of 18 was used, and windows with a difference of 90% were marked as potential unique sequences using an in-house script. In addition, these regions were checked manually (Cole et al., 2014) with Probe Match and Seqmatch at the RDP website and BLAST at NCBI (Johnson et al., 2008).

Primers and probes for qPCR assays were designed from identified unique sequences in using Primer Express software (Applied Biosystems, Foster City, CA, USA) and consulting Agentek Ltd. (Agentek, Tel Aviv, Israel).

### Quantitative Polymerase Chain Reaction

Quantitative polymerase chain reaction assays were performed using the StepOnePlus platform (Applied Biosystems, Foster City, CA, USA), using Universal ABI Mix (Applied Biosystems, Foster City, CA, USA) under the following conditions: 15 min at 95°C, followed by 45 cycles: 15 s at 95°C and 60 s at 60°C. DNA preparations were diluted 1:5 in Ultra Pure Water, PCR grade (Fisher Biotec, Australia) to be further tested in qPCR assays. Duplicate qPCR reactions were carried out in 20 μl final volume in which primer and probe (**Table 1**) concentrations were 500 and 250 nM, respectively.

**Table 1:** Primers and probes of MST qPCR assays used in the study.

Assay	Primers and probe sequence (5′–3′)
Av4143	Av4143F: TGCAAGTCGAACGAGGATTTCT
	Av4143R: TCACCTTGGTAGGCCGTTACC
	Av4143P: [FAM]-AGGTGGTTTTGCTATCGCTTT-[BHQplus]
Av163	Av163F: TCCGGACTACGATGCACTTTC
	Av163R: GCATACAGAGGGAGGCGAAG
	Av163P: [FAM]- AGTTTCGCTCCGTATCGC- [BHQplus]
Av43	Av43F: GCAAGTTGAGCGGAGATATGG
	Av43R: ATCGGCCTATCCCCCAATATA
	Av43P: [FAM]-CTCTTTATATTTTAGCAGCGAACG-[BHQplus]
Av216	Av216F: ATAAGCGAGGGATAACTATTGGAAAC
	Av216R: AACTAGCTAATGCACCGCAGAT
	Av216P: [FAM]-AAGCAACTGTTTCACTTATGGAT-[BHQplus]
Av24	Av24F: GGAAACGACAGCTAATACCGGATA
	Av24R: CTCTTGGCGCATATAGCTTTCA
	Av24P: [FAM]- ATGAGACTTTCGCATGAGAGAC-[BHQplus]
Av13	Av13F: AGTTTGATCCTGGCTCAGGATG
	Av13R: GAGGCAAGTTCCTTACGCGTT
	Av13P: [FAM]- AAGTTACCTTCGGGTAATGAGGAT-[BHQplus]

The insert-vector constructs resulting by the ligation of amplicons into pGEM vectors (Promega, Mannheim, Germany) were used for generating the standard curves. Plasmids were quantified spectrophotometrically, from which the gene copies were calculated. Serial dilutions of cloned pGEM plasmid were carried out independently in triplicates. Plasmid -constructs with the following qPCR amplicons Av13, Av24, Av163, Av216, and Av4143 were sequenced using a commercial sequencing service (HyLabs, Rehovot, Israel).

Sensitivity values of the qPCR assays are presented in percentages and are calculated as the fraction of actual positive [true positive (TP)] host samples divided by all expected positive hosts, including both false negative (FN) and TP, as follows: Sensitivity = TP/(FN + TP).

Specificity values are presented in percentages and are calculated as the fraction of actual negative [true negative (TN)] host samples divided by all expected negative hosts, including both false positive and true negative, as follows: Specificity = TN/(FP + TN).

## Results

The development of MST avian markers included HTS of bacterial 16S rRNA gene from various avian fecal sources. This was followed by two steps of bio-informatic filtering analysis: the first, identified both comparable and distinctive OTUs among the domestic and wild avian fecal sources. The second, compared the16S rRNA gene sequences of the selected OTUs to database of bacteria originating from other host fecal samples, in order to identify unique avian DNA sequences. These analyses were the basis for planning and design of qPCR assays, which were then evaluated to establish a battery of Avian MST assays.

### OTU Analysis of Avian Feces

**FIGURE 1:**
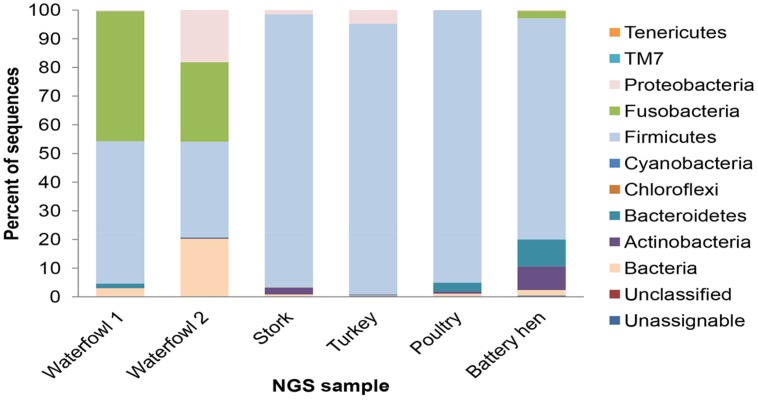
**Taxonomic distribution of associated bacteria of tested fecal avian samples: waterfowl, stork, turkey, poultry, and battery hen given in percents.** Most OTUs were assigned to the phyla of *Firmicutes, Proteobacteria*, and *Fusobacteria*.

All in all, 10 phyla were detected across the tested sample sets, revealing different distribution profiles. Most OTUs were assigned to *Firmicutes, Proteobacteria*, and *Fusobacteria*, and the incidence of other phyla was lower. *Bacteroides* and *Actinobacteria* consist 9.5% of the reads and other phyla (*Verrucomicrobia*, *Tenericutes, Chloroflexi*, and *Cyanobacteria*, and *TM7*) representation was less than 1% each (**Figure 1**). The difference of fecal microbial communities between various tested host species was demonstrated by dissimilar OTU affiliations, as well as dissimilar OTU frequencies. The poultry sample had a relatively homogenous taxonomical profile. Not only were 94% of sequence reads ascribed to the *Firmicutes* phylum, but 90% belong to the *Lactobacillaceae* at the family level (data not shown). 16S rRNA gene read sequences of the turkey sample illustrated a similar but not an identical pattern in which the *Firmicutes* phylum dominated, showing higher diversity at the family level. The bacterial community profiles of the two waterfowl samples overlapped at the identified phyla level but still differed in their relative quantitative representations (**Figure 1**). A high degree of variability was observed in levels of *Proteobacteria, Fusobacteria*, and *Firmicutes* phyla between avian samples. Waterfowl samples had a noticeably high incidence of the *Fusobacteria* phylum, which was almost absent in the domestic avian samples.

### Avian Marker Design

Markers pipeline development included both bioinformatics analyses and laboratory assessment. A total of 59,863 sequence reads were analyzed using a *de novo* OTU generation approach according to the performed sequence pairwise comparisons. This approach resulted in 7205 OTUs, 57 of which were sample group specific. Next, *in silico* analysis for unique DNA was carried out using a dedicated server, http://mst.weizmann.ac.il. Accession numbers of sequences from which qPCR were designed are detailed in **Table 2**.

**Table 2:** Performance features of qPCR assays.

Assay	Taxonomical affiliates	GenBank accession no.	Size (bp)	Slope	Intercept	qPCR efficiency	Range of quantification	Limit of detection (plasmid copies)^a^
Av4143	*Lactobacillus*	LN864462	244	3.75	44.05	84.7	25 – 2.5E8	6
Av163	*Gallibacterium*	LN864463	74	3.3	40.9	100	18 – 3.6E8	9
Av43	*Firmicutes*	LN864464	100	3.39	41.4	97	25 – 2.5E8	6
Av216	Other bacteria	LN864465	122	3.4	40.5	96.8	24 – 2.4E8	6
Av24	*Fusobacteriaceae*	LN864466	76	3.4	40.1	96.8	20 – 2.0E8	5
Av13	*Fusobacteriaceae*	LN864467	119	3.43	42.3	95.6	19 – 3.8E8	8

^a^*Defined as 90% positive detection of qPCR.*

### MST qPCR Assays Characterization

Six out of the nine qPCR assays met performance criteria as evaluated by triplicates of three independent calibration curves of plasmid construct dilutions (**Table 2**). qPCR assays demonstrated similar amplification efficiencies, range of quantification (ROQ) and limit of detection (LOD) defined as 90% positive detection of tested qPCR assays. Amplicon sequencing of Av13, Av24, Av43, Av163, and Av216, showed 100% identity with the corresponding HTS sequences whereas Av4143 had 99% identity.

### Sensitivity and Specificity Assessment

Sensitivity and specificity values were calculated from qualitative analysis of qPCR results. Binary analysis, presence or absence of MST markers, disclosed a variety of marker distribution and incidence in avian hosts (**Table 3**). Av4143 was identified in all avian groups whereas Av163 was totally absent in turkey samples. qPCR profiles of poultry and battery hen samples were similar in that both were positive for Av43 and negative for the three markers Av216, Av24, and Av13. The waterfowl qPCR profile, on the other hand, was a mirror-image of the above, demonstrating no Av43 but showing Av216, Av24, and Av13. Av216, Av24, and Av13 were only detected in waterfowl, and their sensitivity values spanned from 47 to 76%.

**Table 3:** Number of positive detection avian fecal sample in each MST assay (the numbers of total tested samples are specified in parenthesis).

MST marker	Battery hen (50)	Poultry (20)	Turkey (10)	Waterfowl (17)	Sensitivity value (%)
Av4143	48	18	10	14	95^a^
Av163	48	10	0	10	70^a^
Av43	46	18	1	1	91^b^
Av216	0	0	0	13	76^c^
Av24	0	0	0	9	52^c^
Av13	0	0	0	8	47^c^

*Assay sensitivity value was calculated as the percent of true positive detection rate of target tested samples.*

^a^*True positive samples are of avian origin.*

^b^*f True positive samples are of chicken origin.*

^c^*True positive samples are of waterfowl origin.*

Specificity values of qPCR assays were determined by testing non-target fecal and wastewater samples from various origins, including domestic animal and urban wastewater influent samples. The results (**Table 4**) demonstrated relatively high specificity values for most assays, except for the Av163 assay, which was detected in 37% of the bovine samples.

**Table 4:** Number of positive qPCR detections in each avian assay, tested in non avian samples.

Assay	Human wastewater effluent (25)	Bovine (40)	Swine (20)	Horse (9)	Gazelle (10)	Specificity value (%)
Av4143	1	0	1	1	0	97
Av163	0	15	0	0	0	85
Av43	0	1	0	0	0	99
Av216	0	1	0	0	0	99
Av24	0	0	0	0	0	100
Av13	0	0	0	0	0	100

*The numbers of total tested samples are specified in parenthesis. Specificity of qPCR assays calculated as percent of true negative detection rate of non-target samples.*

### Fecal Avian MST Profiles

**FIGURE 2:**
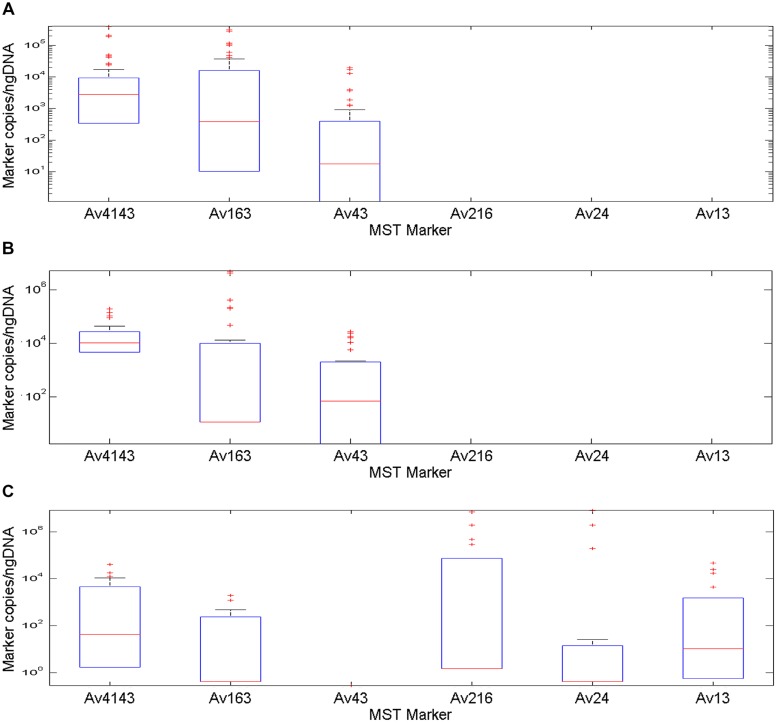
**A box plot diagram demonstrating copy number of MST markers Av4143, Av163, Av43, Av216, Av24, and Av13, identified in fecal samples collected from battery hens, poultry and waterfowls normalized to nano-grams of DNA.** Boxes exhibit 25 and 75 percentiles, lines within boxes represent median values and plus signs identify outliers. **(A)** A marker profile pattern from fecal battery hens revealed occurrence of three different makers: two general avian markers, Av4143 and Av163, and the Av43 poultry and battery hen marker, which was identified at a lower rate. **(B)** The marker profile signature of poultry samples was very similar to that of battery hens. **(C)** An avian marker profile of the waterfowl group comprised five of the six tested markers: Av4143 and Av163, the general avian markers, were identified to a lower extent than they were in battery hens or in poultry. The other three markers showed a wide range of quantification scales.

Quantification qPCR feature was employed to calculate marker copies in fecal samples followed by normalization to DNA mass. The Av4143 marker had the highest median value among MST markers, with a decreasing order between poultry, battery hen and waterfowl fecal samples, respectively (**Figure 2**). Its distribution among the poultry samples demonstrated a restricted 25 and 75 percentile range compared to battery hen and waterfowl samples. Waterfowl profiles included five markers, except the Av43; battery hens and poultry displayed a complex of three markers.

## Discussion

Microbial source tracking notably employs the qPCR method targeting specific bacterial sequences as unique markers of their respective hosts. We aimed to develop additional, useful avian MST assays. A major obstacle was the challenge posed by an abundance of both wild and domesticated avian species, some of which migrate over highly heterogenic environments within relatively short periods of time.

Identification of hosts such as human, bovine, and several avian species, requires targeting of specific sequences, often using the 16S rRNA gene (Marti et al., 2011), by respective qPCR assays. The development of such MST molecular markers traditionally starts with building a fecal microbial library from the desired host. The number of clones within a given library varies greatly between reported studies. For example, the Crane MST marker targeting bacteria related to *C. marimammalium* was designed following analysis of 1,151 16S rRNA gene clones whereas the (Marti et al., 2011) muskrat marker included 62 clones.

The use of HTS for the development of avian MST may enable a comprehensive screening of the microbiome of various avian hosts. Nevertheless, our HTS was performed using samples collected from only six hosts in a single country, albeit from various habitats. This might limit one’s ability to reach conclusions regarding flora from avian populations worldwide.

The taxonomical variability in young poultry and young turkeys was found to be lower than that isolated from battery hens or storks. Phyla identified in avian fecal samples have been previously mapped (Lu et al., 2009), demonstrating a dominance of the *Firmicutes, Proteobacteria*, and *Fusobacteria*. This finding was reaffirmed in the present study where *Firmicutes* had the highest occurrence. The *Bacteriodales* phylum was found in variable frequencies in the tested avian groups, ranging from 0.1% in waterfowl to 9.56% in battery hens. Members of the *Bacteriodales* phylum have not been consistently reported in avian gut and excreta; in some studies (Zhu et al., 2002) they were almost absent; in others (Scupham et al., 2008) they were identified. This variability can be attributed, at least in part, to disparities in study design and to the use of different PCR primer sets.

The HTS approach, which was critical in filtering specific MST sequences, included two stages of bioinformatic analysis. A high correlation was shown between the results of the bioinformatic analysis and the final qPCR confirmation results for some but not all of the assays. The three waterfowl assays targeting Av216, Av24, and Av13, were only found in fecal samples from water dwelling fowls, but not from domestic avian species; this was confirmed by qPCR testing. On the other hand, the two assays, Av4143 and Av163, were found to be qPCR positive in most tested samples; the former was only found in battery hens, and the latter was only found in poultry by QIIME software. These findings might raise concerns regarding the possibility of incomplete representation of specific taxa in the HTS experiment. HTS bias representation of bacterial communities (Zhang et al., 2011) is associated, in part, with library preparation based on 16S rRNA gene PCR amplification of heterogeneous sequences. The qPCR reaction, on the other hand, is designed to amplify specific bacterial community members, and is also characterized by a low LOD. The variations between HTS and qPCR results can be attributed to the differences between their respective abilities to detect low copy number sequences.

Simultaneous screening of multiple sequences at the HTS approach allowed identification of an array of assays for various avian groups. The sensitivity values ranged from 47 to 95%; the lower values ascribed primarily to the waterfowl assays and did not meet the 80% benchmark (Boehm et al., 2013). This demonstrates some of the limitations of studying a single geographic location, when testing only a limited sample size of waterfowl. A broader validation (Reischer et al., 2013) in different geographical locations will provide a more reliable sensitivity value, as previously reported.

Specificity values were all above 85% and indicated that the bioinformatic sorting process pinpoints unique determinants of specific bacteria species in avian feces.

Environmental contamination sources can be better understood using MST. The reliability and power of MST increase with the number of available assays which can be employed in evaluation of avian fecal bacteria. As a result, resolution and understanding are more attainable with simultaneous use of an array of markers.

## Author Contributions

Analysis using Qiime, OTU analysis, co-authorship, and critical manuscript revision: BD, VC-C; Putting together a process for seeking unique determinants and constructing a unique, 16S rRNA gene sequence database of fecal origin, followed by creation of free on-line access to this database, co-authorship and critical manuscript revision: SB-D, JP; conception and design of the work including all stages thereof, acquisition, analysis, and interpretation of all data, co-authoring the work, critically revising it, and approving the final submitted version: ER, SO, SB-D, JP, VC-C, BD, VK, YK; agreement to be accountable for all aspects of the work: BD, ER, JP, SB-D, SO, VC-C, VK, YK.

## Conflict of Interest Statement

The authors declare that the research was conducted in the absence of any commercial or financial relationships that could be construed as a potential conflict of interest.
